# HCV-infected individuals have higher prevalence of comorbidity and multimorbidity: a retrospective cohort study

**DOI:** 10.1186/s12879-019-4315-6

**Published:** 2019-08-23

**Authors:** Curtis L. Cooper, Chrissi Galanakis, Jessy Donelle, Jeff Kwong, Rob Boyd, Lisa Boucher, Claire E. Kendall

**Affiliations:** 1 Department of Medicine, University of Ottawa, Ottawa, Canada; 2 Clinical Epidemiology Program, Ottawa Hospital Research Institute, The Ottawa Hospital-General Campus, G12-501 Smyth Rd, Ottawa, Ontario K1H8L6 Canada; 3 ICES, Toronto, Canada; 4 Department of Family and Community Medicine, University of Toronto, Toronto, Canada; 5 Dalla Lana School of Public Health, University of Toronto, Toronto, Ontario Canada; 6 Sandy Hill Community Health Centre, Ottawa, Canada; 7 Bruyère Research Institute, Ottawa, Canada; 8 School of Epidemiology and Public Health, University of Ottawa, Ottawa, Canada; 9 Department of Family Medicine, University of Ottawa, Ottawa, Canada

**Keywords:** HCV, Comorbidity, Multimorbidity, Direct acting antiviral

## Abstract

**Background:**

Almost 1% of Canadians are hepatitis C (HCV)-infected. The liver-specific complications of HCV are established but the extra-hepatic comorbidity, multimorbidity, and its relationship with HCV treatment, is less well known. We describe the morbidity burden for people with HCV and the relationship between multimorbidity and HCV treatment uptake and cure in the pre- and post-direct acting antiviral (DAA) era.

**Methods:**

We linked adults with HCV at The Ottawa Hospital Viral Hepatitis Program as of April 1, 2017 to provincial health administrative data and matched on age and sex to 5 Ottawa-area residents for comparison. We used validated algorithms to identify the prevalence of mental and physical health comorbidities, as well as multimorbidity (2+ comorbidities). We calculated direct age- and sex-standardized rates of comorbidity and comparisons were made by interferon-based and interferon-free, DAA HCV treatments.

**Results:**

The mean age of the study population was 54.5 years (SD 11.4), 65% were male. Among those with HCV, 4% were HIV co-infected, 26% had liver cirrhosis, 47% received DAA treatment, and 57% were cured of HCV. After accounting for age and sex differences, the HCV group had greater multimorbidity (prevalence ratio (PR) 1.38, 95% confidence interval (CI) 1.20 to 1.58) and physical-mental health multimorbidity (PR 2.71, 95% CI 2.29–3.20) compared to the general population. Specifically, prevalence ratios for people with HCV were significantly higher for diabetes, renal failure, cancer, asthma, chronic obstructive pulmonary disease, substance use disorder, mood and anxiety disorders and liver failure. HCV treatment and cure were not associated with multimorbidity, but treatment prevalence was significantly lower among middle-aged individuals with substance use disorders despite no differences in prevalence of cure among those treated.

**Conclusion:**

People with HCV have a higher prevalence of comorbidity and multimorbidity compared to the general population. While HCV treatment was not associated with multimorbidity, people with substance use disorder were less likely to be treated. Our results point to the need for integrated, comprehensive models of care delivery for people with HCV.

**Electronic supplementary material:**

The online version of this article (10.1186/s12879-019-4315-6) contains supplementary material, which is available to authorized users.

## Background

Approximately 1% of Canadians are hepatitis C (HCV)-infected [1, 2]. Among a wide range of infectious diseases, HCV confers a greater burden with respect to premature mortality and reduced functioning [3]. The liver-specific complications of HCV, including liver failure and hepatocellular carcinoma, are well established, as are the benefits of curative HCV antiviral therapy on these outcomes [4]. In contrast, the burden of associated extra-hepatic comorbidity is less well known. Several studies have established the association of HCV with chronic kidney disease and renal impairment [5–10], depression [11–13], neurological disorders [14] and malignancies [15, 16], whereas the evidence is less consistent for association with diabetes mellitus [7, 17–19] and cardiovascular disease [17, 20–22]. With other comorbidities, the association is related to common risk factors including smoking, excess alcohol use, recreational drug use, and poverty [23, 24]. To date, there are a limited number of studies that have comprehensively evaluated comorbidity among people with HCV. For example, Stasi et al. [25] used a study-specific clinical database for patients from 16 hospitals in Tuscany to look at comorbidities amongst HCV patients, Sicras-Mainar et al. [26] conducted a similar study based on medical records at 8 primary care centres in Spain, and Lauffenburger et al. [27] used a US commercial claims database for the same purpose. These studies showed significant comorbidities such as diabetes, dyslipidaemia and hypertension amongst HCV patients, but did not report on multiple comorbidities. Liu et al. [28] reported on multiple comorbidities in their study of Taiwanese adult HCV patients using a physician-completed survey, as did Ruzicka et al. [29] in their study of Japanese adult HCV patients using a medical claims database and Louie et al. [30] in their study of patients with chronic HCV using a US medical claims database. All of these studies showed a significant prevalence of multiple comorbidities (e.g., more than 50% of Japanese HCV patients had ≥4 comorbidities, and 52% of US patients reported 6–15), with the latter two showing this to be greater than amongst matched controls (e.g., only 29.5% of Japanese non-HCV patients had ≥4, and over 47% of US patients reported ≤5 comorbidities). However, these studies either did not use validated or standardized ascertainment methods, or the diagnoses focused on were ill-defined.

In the past, both mental health and physical health comorbidity, and their multimorbidity, may have hindered HCV treatment with interferon and ribavirin-based therapy given the long duration of treatment, the need for weekly subcutaneous injections, and the heavy burden of side effects and toxicities [31, 32]. More recently, Direct Acting Antivirals (DAA) have provided the opportunity to treat people in much less time, with next to no side effects, and with cure rates that are well over 90% [33–36]. However, whether multimorbidity remains a barrier to treatment initiation and successful antiviral treatment outcome in the DAA era is unclear. In two cohort evaluations of DAA recipients, the presence of comorbidities predicted diminished antiviral treatment outcomes [37, 38].

In this study, we uniquely used diverse HCV clinical cohort data linked to robust health administrative databases from a universal, single-payer healthcare system. We used these data to comprehensively i) describe the prevalence of physical and mental health comorbidities and their multimorbidity in a cohort of people living with HCV; ii) compare this prevalence to an age-, sex-, and geographically-matched population without HCV; iii) examine the association between multimorbidity and HCV treatment with interferon- and DAA-based antiviral therapy initiation, and; iv) among those treated with DAA, examine the association between multimorbidity and cure. The role of comorbid substance use on HCV antiviral treatment uptake and outcome use was specifically assessed.

## Methods

### Study design

This was a retrospective cohort study of patients with HCV at The Ottawa Hospital Viral Hepatitis Program (TOHVHP).

### Data sources

We used data from TOHVHP, a publicly funded, academic tertiary care-based regional referral centre for viral liver disease care in Ottawa, Canada. This is the primary referral centre for HCV in Eastern Ontario, irrespective of stage of disease. The cohort consists of demographic, clinical, and laboratory data collected from consenting participants who receive care in TOHVHP clinics (Ottawa Health Science Network Research Ethics Board: 2004–196). Participants agreed to linkage and analysis of non-nominal data.

We linked TOHVHP data to provincial health administrative data at ICES, an independent non-profit research institute whose legal status under Ontario’s health information privacy laws allows it to collect and analyze health care and demographic data, without consent, for health system evaluation and improvement. The following ICES datasets were linked using unique encoded identifiers and analyzed at ICES: the Registered Persons Database, an electronic registry of demographic and mortality data for residents eligible for provincial health care; the Ontario Health Insurance Program (OHIP) billing claims dataset, which captures services provided by about 95% of physicians in Ontario; the Discharge Abstract Database for all hospital admission and discharge data; the Ontario Mental Health Reporting System for all admissions to designated mental health beds; CONTACT (eligibility summaries and yearly health services contact); the Ontario Cancer Registry for data on patients with diagnoses of cancer; and the Immigration, Refugee and Citizenship Canada (IRCC) Permanent Resident database, which includes the country of birth of immigrants who landed in Ontario as of 1986. We also used 2011 Statistics Canada Census data to generate neighborhood income quintiles by linking postal code of residence to the mean household income.

### Participants

TOHVHP and ICES datasets were linked either deterministically, using unique, encoded identifiers derived from participants’ OHIP numbers, or probabilistically, based on a number of other personal identifiers (first and last name, date of birth, sex, postal code). We included all TOHVHP patients with a first visit from April 1, 2000 to the index date of April 1, 2017. We excluded participants for the following reasons: less than age 18 years at index, invalid OHIP card at index, death date in ICES data before index date, invalid sex or age in ICES data, missing or out-of-province postal code, or not seen by the program in the 5 years prior to index date.

To compare TOHVHP participants to the general Ontario population, we randomly selected control individuals, matched on age, sex, and public health unit, in a 5:1 ratio.

### Outcomes

To ascertain comorbidities, we used validated algorithms developed at ICES to determine the prevalence of the following chronic conditions: acute myocardial infarction (AMI) [39], asthma [40], congestive heart failure (CHF) [41], chronic obstructive pulmonary disease (COPD) [42], dementia [43], diabetes [44], hypertension [45], HIV [46] and rheumatoid arthritis [47]. As with other multimorbidity studies [48, 49], for conditions where a derived ICES cohort did not exist, we adopted a similar approach (i.e. the presence of any one inpatient hospital diagnostic code (DAD data) or two or more outpatient physician billing codes (OHIP data) within a 2 year period using relevant ICD-9 and ICD-10 codes) to define the following chronic conditions: cardiac arrhythmia, osteoarthritis, osteoporosis, renal failure, and stroke, as well as for mental health conditions and substance use disorders. (Appendix 1 in Table 3) [48–54]. We determined the prevalence of any cancer using the OCR. Liver failure and liver transplant were ascertained if one ICD9 or ICD10 code was billed in OHIP or DAD in the previous 10 years. (Appendix 1 in Table 3).

We used disease count to measure the prevalence of multimorbidity [55]. Physical multimorbidity was defined as the presence of two or more listed physical chronic conditions, and physical-mental health multimorbidity was defined as a combination of both at least one mental health condition and at least one physical chronic health condition. Among patients in the HCV group, this multimorbidity is in addition to HCV as their index condition.

### Variables

We used the following sources to examine cohort characteristics: (1) RPDB: age, sex; (2) IRCC: immigration status, categorized as immigrant from a country with a generalized HCV epidemic, immigrant from a non-HCV endemic country, or a long-term resident, as IRCC files date back only to 1985. Countries with intermediate high to high prevalence (rates greater than 1.3% based on HCV serology or viremia criteria) were considered HCV-endemic countries [56, 57]; (3) TOHVHP: time from HCV diagnosis, HCV treatment status, categorized as treated with a DAA, an interferon-based regimen, or neither. Any interferon recipient, irrespective of co-administered RBV and/or DAA was classified in the interferon grouping. Only those on interferon-free regimens were classified as DAA recipients. Cure was determined by the presence of at least one blood test demonstrating the absence of HCV RNA at least 12 weeks following completion of HCV antiviral therapy.

### Statistical analyses

We used descriptive statistics to describe the demographic and clinical characteristics, and compared the groups using two-sample t-tests for continuous variables and chi-squared tests for categorical variables. We calculated the prevalence of individual physical and mental health comorbidities, physical multimorbidity, and physical-mental health multimorbidity. We used direct standardization to calculate age- and sex-standardized prevalence rates using the Canadian 2011 population as the reference standard. We estimated the comparative prevalence ratios of these rates, with 95% confidence intervals calculated using the formula provided by Breslow and Day [58]. We also compared the prevalence of treatment with interferon and DAA among those with and without multimorbidity by age group and the prevalence of cure among those treated with DAA by age group. We further stratified these associations for both men and women. Finally, given the anticipated prevalence of substance use disorders in our population, we also stratified treatment and cure prevalence among those specifically with and without substance use disorders. We defined statistical significance a priori at a *p*-value of 0.05. All statistical analyses were performed using SAS version 9.4 (SAS Institute, Cary, North Carolina).

## Results

Most TOHVHP patients (90.4%) were successfully linked to ICES data. After exclusions, our HCV group comprised 1209 individuals (Additional file 1: Figure S1). The demographic and clinical characteristics of the HCV group and the matched general population (*n* = 6045) are shown in Table 1. The mean age was 54.5 years and 64.7% were male. In both groups, approximately 13% of participants had immigrated to Canada. Among the HCV group, 4.4% were HIV co-infected and 0.5% were hepatitis B virus co-infected. Of those with known fibrosis stage data, 33.2% (309/932) were cirrhotic. Approximately one-quarter (27.2%) were HCV antiviral treatment naïve, 4.2% had been treated without achieving SVR, 57.1% had received treatment and achieved a cure, and 11.4% had missing treatment data. Among those treated, 76.0% received DAA-containing regimens. Compared to the general population, HCV-infected individuals had significantly greater comorbidity, and had higher prevalence of multimorbidity (29.9% vs 20.8%, *p* < 0.001) and physical-mental health multimorbidity (24.7% vs 9.5%, *p* = 0.001).

**Table 1:** Demographic and clinical characteristics of TOHVHP HCV group and the general population

Variable	TOHVHP HCV group	General population	*p*-value
	*N* = 1209	*N* = 6045	
	N(%)	N(%)	
Age (yrs) (mean, SD)	54.54 ± 11.42	54.54 ± 11.41	1.00
Age (yrs) (median, IQR)	56 (49–62)	56 (49–62)	1.00
Age category			
16–25	15 (1.2%)	75 (1.2%)	1.00
26–35	77 (6.4%)	385 (6.4%)	
36–45	127 (10.5%)	635 (10.5%)	
46–55	369 (30.5%)	1845 (30.5%)	
56–65	464 (38.4%)	2320 (38.4%)	
66–75	122 (10.1%)	610 (10.1%)	
75+	35 (2.9%)	175 (2.9%)	
Sex			1.00
Male	782 (64.7%)	3910 (64.7%)	
Female	427 (35.3%)	2135 (35.3%)	
Immigration status			0.49
Canadian citizen	1056 (87.3%)	5290 (87.5%)	
Immigrant from HCV endemic country	54 (4.5%)	230 (3.8%)	
Immigrant from non-endemic country	99 (8.2%)	525 (8.7%)	
HCV mono-infection	1151 (95.2%)	–	
HCV-HIV co-infection	53 (4.4%)	–	
HCV-HBV co-infection	6 (0.5%)	–	
Time from HCV diagnosis			
< 5 years from index date	124 (10.3%)	–	
5 to < 10 years from index date	123 (10.2%)	–	
10+ years from index date	263 (21.8%)	–	
Missing	699 (57.8%)	–	
Treatment status^a^			
Treated and cured	690 (57.1%)	–	
Treated and not cured	51 (4.2%)	–	
Never treated	329 (27.2%)	–	
Missing	139 (11.4%)		
HCV treatment type (*n* = 741)			
Direct acting antiviral (DAA)	563 (76.0%)	–	
Interferon (IFN)	178 (24.0%)	–	
Highest fibrosis score			
1	332 (27.5%)	–	
2	171 (14.1%)	–	
3	120 (9.9%)	–	
4	309 (25.6%)	–	
Missing	277 (22.9%)	–	
Prevalence of multimorbidity (at least two physical conditions)	361 (29.9%)	1259 (20.8%)	< 0.001
Prevalence of physical-mental health multimorbidity	299 (24.7%)	574 (9.5%)	< 0.001

^a^ 139 (11.5%) individuals could not be classified into any of the treatment status categories. Of these 33 (2.7%) were treated but had not completed their post-treatment bloodwork and cured status could not be determine, 72 (6.0%) were potentially LTFU, and 34 (2.8%) were unaccounted for (not found in either treatment database, but no record of lab test results (VL bloodwork))

Table 2 presents the age- and sex-standardized prevalence rates for the two groups, along with the prevalence rate ratios and 95% confidence intervals. The prevalence in the HCV group was significantly higher for asthma (prevalence ratio (PR) 1.32; 95% confidence interval (CI) 1.06 to 1.64), any cancer (PR = 1.76; 95% CI, 1.22 to 2.55), COPD (PR = 2.42; 95% CI 1.73 to 3.39), diabetes (PR = 1.32; 95% CI 1.06 to 1.64), and renal failure (PR = 2.92; 95% CI 1.90 to 4.48), but not for cardiac outcomes (arrhythmias, congestive heart failure, chronic coronary syndrome). The burden of all mental health conditions including non-psychotic mood and anxiety disorders, other mental health illnesses, and substance use were significantly more prevalent in the HCV group (PR = 2.22; 95% CI 1.86 to 2.64, PR = 2.71; 95% CI 2.06 to 3.55, and PR = 26.50; 95% CI 18.35 to 38.27, respectively). Liver failure was significantly more prevalent in the HCV group (PR = 6.63; 95% CI 2.72 to 11.81). The number of liver cancers and liver transplants were greater in the HCV group, with 30 and 15 affected participants respectively, but numbers in the comparison population were too small to calculate prevalence ratios. Finally, multimorbidity and combined physical-mental health comorbidity were significantly more prevalent in the HCV group (PR = 1.38; 95% CI 1.38 to 1.58 and PR = 2.71; 95% CI 2.29 to 3.20, respectively).

**Table 2:** Comorbidity and multimorbidity prevalence between TOHVHP HCV group and the general population (age and sex standardized to 2011 Ontario population)

Condition	TOHVHP HCV group		General population		HCV:General popn
	*N* = 1209		*N* = 6045		Prevalence ratio (95% CI)
	n	Prevalence (%)	n	Prevalence (%)	
Acute myocardial infarction^a^	< 6	–	< 17	–	–
Asthma	206	19.40	746	14.74	**1.32 (1.06–1.64)**
Cancer (any)*	83	7.78	241	4.42	**1.76 (1.22–2.55)**
Cardiac arrhythmia	31	2.60	258	4.19	0.62 (0.37–1.06)
Chronic obstructive pulmonary disease*	97	7.78	137	3.22	**2.42 (1.73–3.39)**
Congestive heart failure*	46	3.79	122	3.14	1.21 (0.77–1.88)
Chronic coronary syndrome	89	5.61	422	5.79	0.97 (0.68–1.38)
Dementia^a^	< 13	–	48	–	–
Diabetes	207	13.27	819	10.07	**1.32 (1.06–1.64)**
Hypertension	364	24.97	1746	23.14	1.08 (0.91–1.27)
Non psychotic mood and anxiety disorders	330	31.24	805	14.08	**2.22 (1.86–2.64)**
Other mental illness	157	13.35	308	4.93	**2.71 (2.06–3.55)**
Osteoporosis	35	3.11	157	3.35	0.93 (0.55–1.58)
Rheumatoid arthritis	22	1.19	55	0.97	1.23 (0.67–2.24)
Renal failure*	87	6.19	97	2.12	**2.92 (1.90–4.48)**
Stroke*	22	1.72	101	1.83	0.94 (0.49–1.81)
Substance use disorder*	287	32.83	88	1.24	**26.50 (18.35–38.27)**
Hepatocellular cancer^a^	30	–	<=6	–	–
Liver failure*	71	4.29	38	0.65	**6.63 (3.72–11.81)**
Liver transplant^a^	15	–	0	–	–
Multimorbidity (at least two physical conditions)	554	39.20	2053	28.48	**1.38 (1.20–1.58)**
Physical-mental health multimorbidity	396	32.24	792	11.91	**2.71 (2.29–3.20)**

^a^ indicate that direct standardization could not be performed due to sparse age-sex cell sizes. In such cases, direct standardization was performed

Note: Prevalences were standardized using the **2011** Ontario Population, the n is the true size from each group

**Bold** faced font indicates statistically significant at the 0.05 level

*Significant Finding

**Fig. 1:**
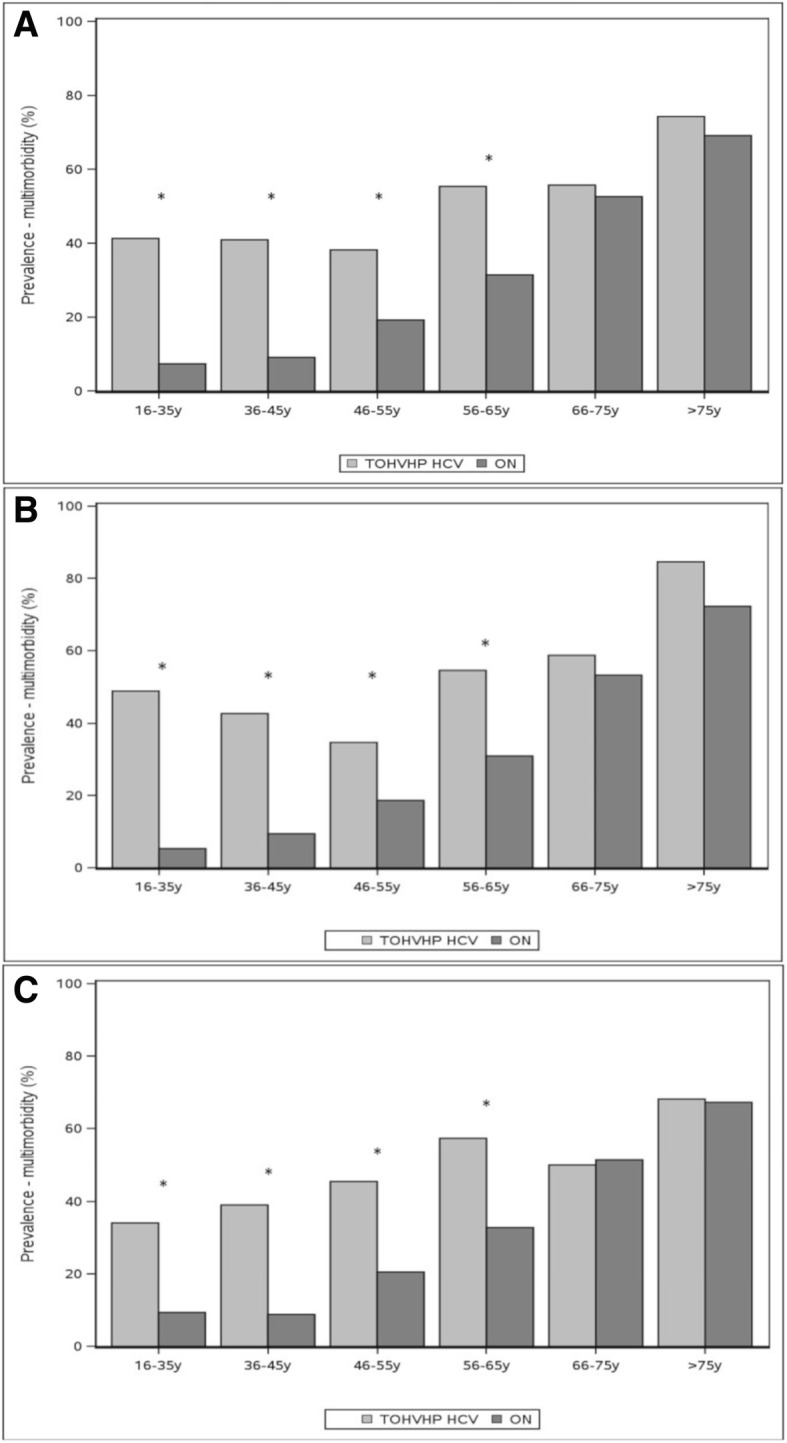
**a** Multimorbidity prevalence (> = 2 conditions) by age group among men and women in the TOHVHP HCV group (*n* = 1209) versus the general population (*n* = 6045). **b** Multimorbidity prevalence (> = 2 conditions) by age group among men in the TOHVHP HCV cohort (*n* = 782) versus the Ontario cohort (*n* = 3910). **c** Multimorbidity prevalence (> = 2 conditions) by age group among women in the TOHVHP HCV cohort (*n* = 427) versus the Ontario cohort (*n* = 2135). * *P* < 0.05

Figure 1a shows the prevalence of multimorbidity by age category for both cohorts. Multimorbidity was significantly greater among HCV patients for all age groups until age 66 years, with differences most striking in the early age categories. This trend persisted when the groups were stratified by sex (Fig. 1b and c).

**Fig. 2:**
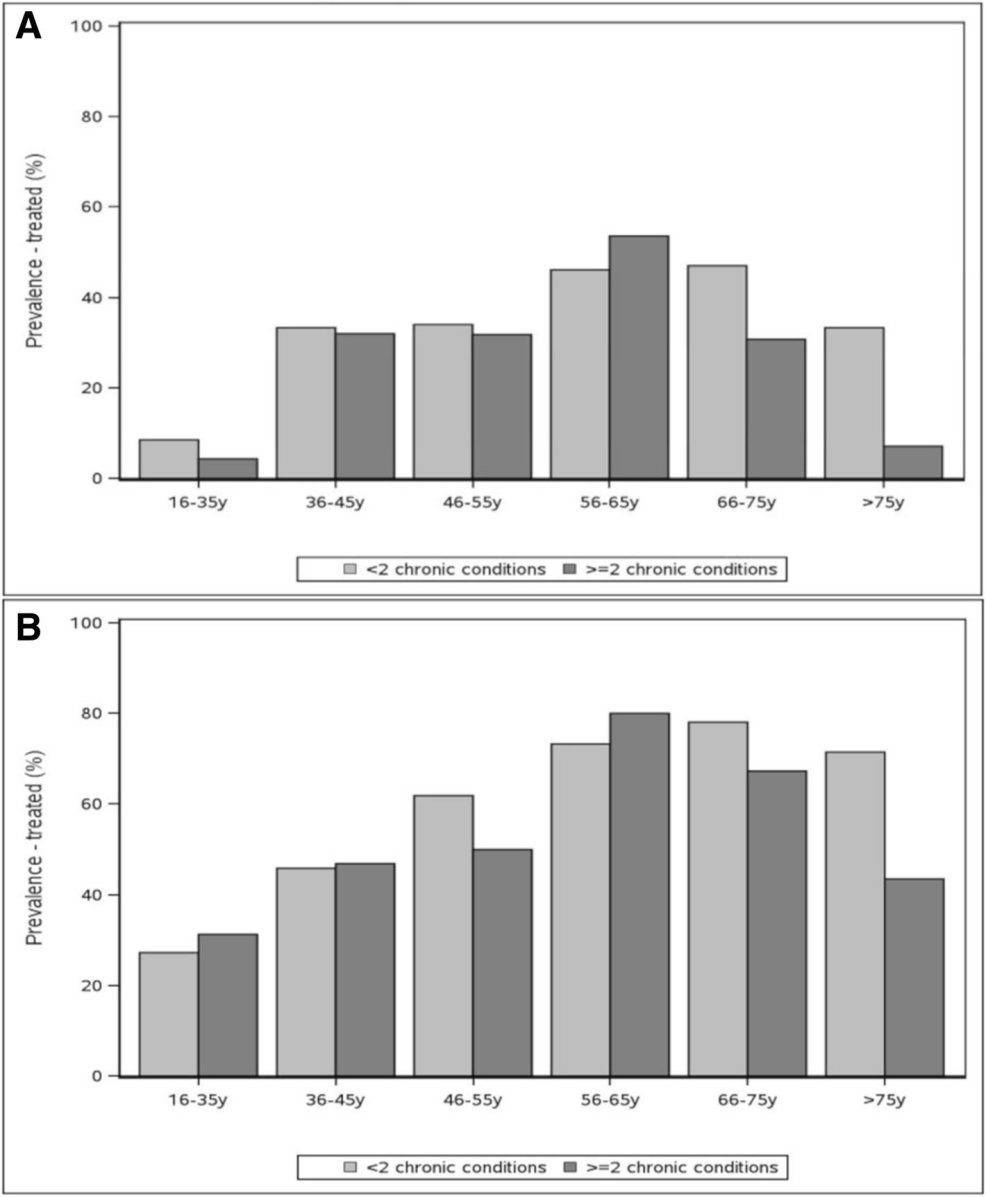
**a** Treatment prevalence by age group and multimorbidity (> = 2 conditions) among participants in the TOHVHP HCV cohort – Treated with interferon (*n* = 178). **b** Treatment prevalence by age group and multimorbidity (> = 2 conditions) among participants in the TOHVHP HCV cohort – Treated with DAA (*n* = 563)

Figure 2a and b show the prevalence of treatment with interferon and DAA among participants with and without multimorbidity, by age group. Treatment prevalence did not significantly vary by whether the individual had multimorbidity at any age among either the 178 people treated with interferon or the 563 people treated with DAA. These trends generally held when the group was stratified by age, although among those treated with interferon, compared to those without multimorbidity, statistically significantly more men aged 56–65 years were treated (63.2% vs 42.6%, *p* = 0.03, data not shown).

**Fig. 3:**
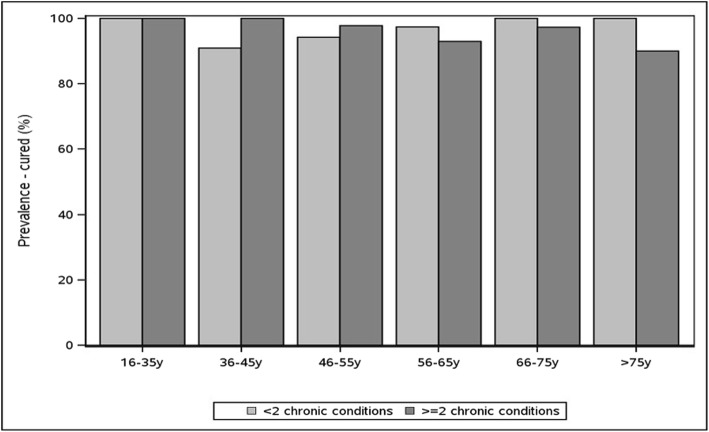
Cured prevalence by age group and multimorbidity (> = 2 conditions) among participants treated with DAA in the TOHVHP HCV cohort (*n* = 563)

Figure 3 shows the prevalence of cure among participants treated with DAA among people with HCV with and without multimorbidity, by age group. Among the 563 people treated with DAA, 95.6% (*n* = 538) were cured, and cure from DAA treatment did not differ among those with and without multimorbidity. This trend held when the group was stratified by sex (data not shown). Note that small cell sizes made estimates difficult for some age groups.

**Fig. 4:**
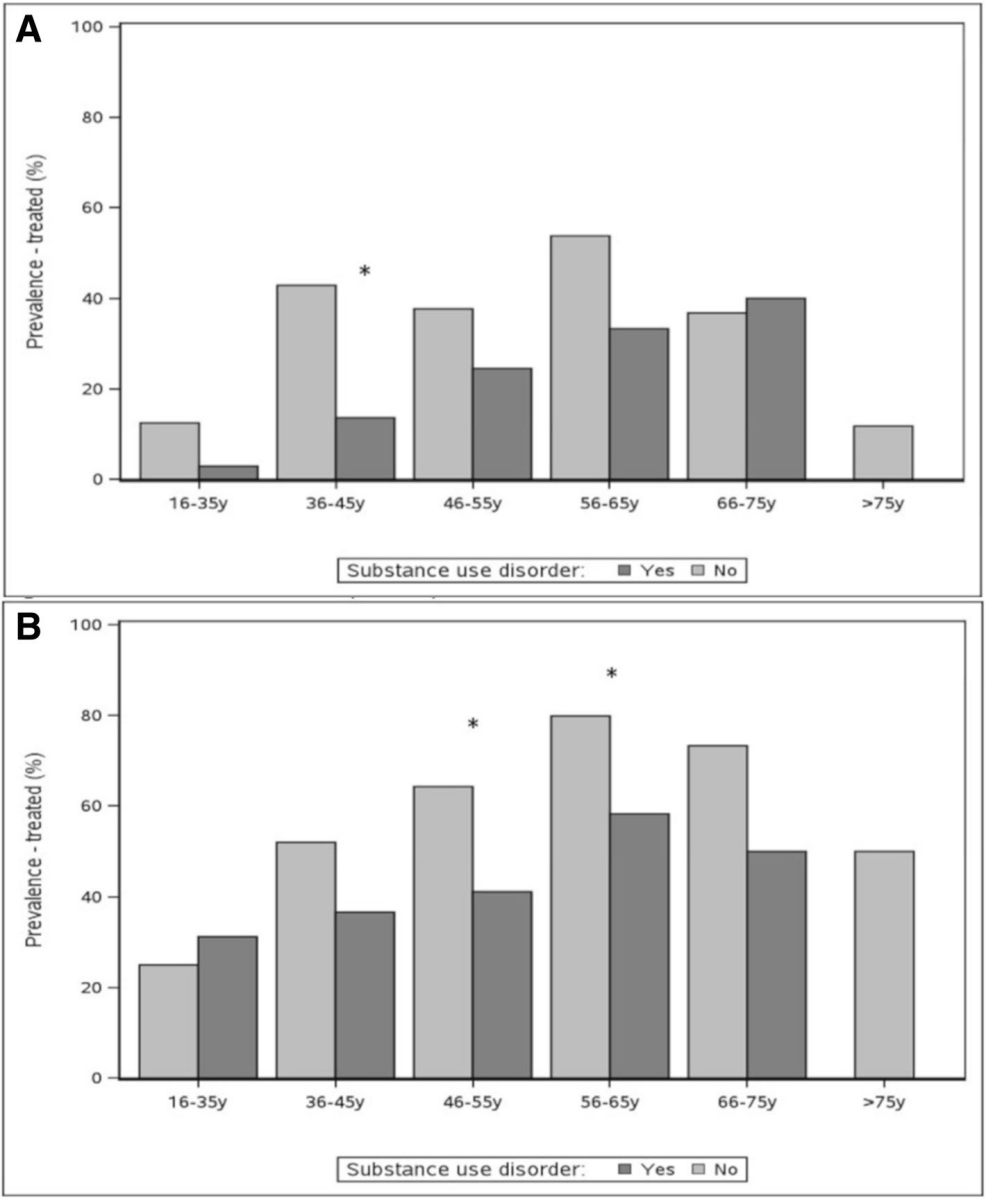
**a** Treatment prevalence by age group among participants with and without substance use disorder in the TOHVHP HCV cohort – Treated with IFN (*n* = 178) **b** Treatment prevalence by age group among participants with and without substance use disorder in the TOHVHP HCV cohort – Treated with DAA (*n* = 563)

Figure 4a and b show the prevalence of treatment with interferon and DAA among people with HCV with and without substance use disorders, by age group. Among the 178 people treated with interferon, treatment prevalence generally did not significantly vary by whether the individual had substance use disorder except among individuals aged 36 to 45 years, among whom a lower proportion with substance use disorder received treatment treated (13.6% vs 42.9%, *p* = 0.02). Among the 563 people treated with DAA, treatment prevalence was significantly lower among those with substance use disorder aged 46 to 55 years and 56 to 65 years (41.1% vs 64.3%, *p* = 0.001 and 58.3% vs 79.9%, *p* = 0.003, respectively).

**Fig. 5:**
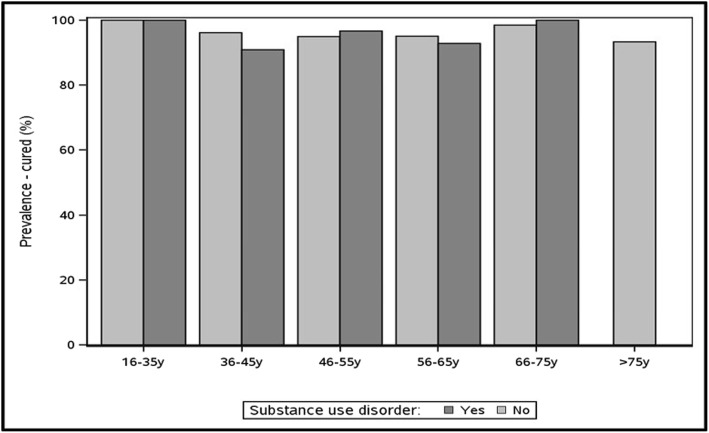
Cured prevalence by age group among participants with and without substance use disorder treated with DAA in the TOHVHP HCV cohort (*n* = 563)

Figure 5 shows the prevalence of cure among participants with and without substance use disorder treated with DAA. Among the 563 individuals treated with DAA, the proportion of those cured did not differ among those with and without substance use disorder. Note that small cell sizes made estimates unstable for some age groups (data not shown).

## Discussion

In our study of a clinical cohort linked to health administrative data with validated chronic disease ascertainment algorithms, we found individual comorbidity burden to be greater among people with HCV than the general population. This was especially true for substance use, mental health, and liver-related diseases. In addition, we found that the multimorbidity of these conditions was significantly greater for people with HCV, including both physical and combined physical and mental health multimorbidity. This finding was most significant among younger age groups and persisted up until age 66 years for both men and women. We also found that multimorbidity was not associated with receiving interferon-containing or DAA treatment, nor with achieving cure with DAA treatment. However, people with substance use disorder in their middle age were less likely to receive treatment even with DAA. As there is no known validated algorithm for HCV ascertainment using administrative data, our study is the first to our knowledge to use combined cohort and population-based data to determine multimorbidity among a cohort of people living with HCV and to compare this to a general, representative population.

We found an increase in the prevalence of several chronic conditions among people living with HCV. Substance use is a well-established risk factor for HCV exposure and the prevalence of concomitant mental health conditions is recognized as being very high in those with HCV infection [59]. However, we found even higher prevalence of these conditions in our cohort than other populations. For example, we found substance use disorders among nearly one-third of the HCV patients in our cohort, whereas other studies found 4–25% prevalence. Similarly we found mood and anxiety disorders among one-third of HCV patients, compared to 13.9–23.7% for depression in other studies [26, 27, 30]. One reason for this could be that our setting has universal access to care, which could have facilitated patient access for these conditions. In addition, the consequences of chronic HCV infection include liver failure, hepatocellular carcinoma, and the need for liver transplantation [60]. As such, our finding that liver-related disease is higher in those living with HCV compared to the general population is not surprising.

We also found that the prevalence of several non-hepatic conditions was greater among people with HCV. This was true for conditions known to have causal relationships with HCV, including diabetes, some cancers, and chronic renal failure [5–10, 15–19], although our estimates for these conditions were generally lower than those in other studies with less stringent, unvalidated methods of chronic disease ascertainment [26, 27, 29, 30] or from clinical charts [25]. For example, we found the prevalence of diabetes in our cohort was 13.27%, compared to 13.8–26.1% in other studies [25–27, 29, 30]. We also found greater prevalence of lung conditions including asthma and COPD, which were, when combined, higher than some [26, 29] and lower [27] than prevalences reported in studies with less robust definitions, likely due to overlap between these conditions and their relationship with smoking. We also did not find evidence of increased prevalence of any cardiovascular conditions among people with HCV compared to the general population, including both acute (myocardial infarction, stroke) or chronic (hypertension, chronic coronary syndrome, congestive heart failure, arrhythmia) conditions. This is in contrast to other studies that found increased prevalences of these conditions among people with HCV [26–29], although, again, our condition ascertainment was more rigorous. For example, we found hypertension among one quarter of our HCV patients, whereas other studies reported prevalence of 31.4–40.1% [26, 27, 29, 30]. Again, we note that the comparator studies used a variety of ascertainment methods for defining comorbidity, the majority of which have not been validated, or included symptom-based diagnoses such as pain, making comparisons challenging.

Importantly, we also found that the multimorbidity of these conditions, including combined physical and mental health multimorbidity, was also particularly high in those living with HCV infection. To our knowledge, only one other study has quantified this burden [30]: these authors found slightly greater proportions of multiple conditions. However, ascertainment of these conditions was not validated and many of those conditions, including those at greatest prevalence contributing to multimorbidity counts, were symptom-based, such as abdominal pain, back problems, and fatigue, thus may not have the same implications for engaging people in care and treatment.

In our study, multimorbidity was not associated with initiation of either interferon or DAA regimens. This result was surprising, as interferon is well known for producing and/or exacerbating underlying medical and mental health conditions, many of which have been considered relative or absolute contraindications to initiating interferon-based therapy [61]. We suspect this reflects our practice of reserving interferon-based treatment to those with more advanced liver fibrosis, who would have been older and more likely to have multimorbidity as demonstrated by our analysis. A major advantage of DAA therapy is that there are few contraindications, even in those with advanced liver disease or other comorbidities [62], which supports our finding that multimorbidity was not associated DAA-based treatment initiation. While, in at least one study, early discontinuation of interferon-free therapy was predicted in part by comorbidity burden [38], in our analysis the proportion of DAA-treated patients achieving a SVR was unrelated to prevalence of multimorbidity. Finally, when we restricted our analyses to people with substance use disorders, those who were middle-aged were less likely to receive DAA, although these individuals were equally likely to achieve cure. Others in similar settings have also noted this disparity [63]. This finding is of concern given that DAA therapy is reimbursed by our provincial formulary for nearly all patients and that the average age of people with HCV who use drugs in Canada is in the mid to late forties range [63, 64]. It is critical to develop policies and strategies that facilitate DAA uptake and completion in those facing barriers to treatment [65, 66].

A strength of our study is that we used a combination of rich data from a diverse cohort of people living with HCV in a broad geographic region combined with robust population-level data for comorbidity ascertainment. In addition, recognizing that mental health comorbidity, in particular substance use disorder, may be particularly high among people living with HCV, we stratified our findings to include those with and without these conditions so as not to overestimate the impact of their prevalence on all people living with HCV. Nevertheless, there are limitations. Although we used validated algorithms to identify comorbidities, our estimates are restricted to people who are diagnosed and receiving care. We also could not ascertain treatment outcomes among people who were lost to follow-up from care, and in particular among interferon-based treatment recipients for whom treatment outcomes were incomplete. This would introduce bias in our results if the multimorbidity of those lost to follow up had significantly different morbidity from those who were retained for analysis. Evaluation of comorbidity as a function of liver fibrosis stage would have been revealing. However, our dataset did not allow for this analysis. Finally, our study setting is one of single payer, universal care, which likely optimizes disease ascertainment based on diagnosis codes but may not be generalizable to other settings. Given the retrospective nature of this analysis we were able to identify associations but not establish causality.

People living with HCV have a higher prevalence of many comorbidities as well as both physical and physical-mental health multimorbidity compared to the Ontario population. Middle-aged individuals with substance use disorder were less likely to receive treatment, even in the DAA era. In addition to well-known reductions in mortality and liver-specific comorbidity, HCV treatment has also been shown to reduce future comorbidity, including diabetes, renal disease, cardiovascular disease, mental and cognitive health, and quality of life [67–73]. As such, our findings support current calls for taking a broad, inclusive approach to offering HCV antiviral therapy regardless of physical and mental health comorbidity and mode of HCV transmission, including injection drug use. With the evidence that the management of chronic diseases is most effectively and economically provided in well-supported primary care settings [74, 75], our findings call for integrated, comprehensive, community-oriented approaches to HCV care delivery. Specific strategies may include involving peers in care, case management, integration of HCV care with substance use, social service delivery, primary care services [76], use of telehealth services [77], and self-management strategies [78–80].

## Conclusions

People with HCV have a higher prevalence of comorbidity and multimorbidity compared to the general population. While HCV treatment was not associated with multimorbidity, people with substance use disorder were less likely to be treated. Given the capacity for treatment to prevent and mitigate the effects of comorbidity among people with HCV, our results point to the need for integrated, comprehensive models of care delivery for people with HCV with a particular emphasis on mental health and addictions care.

### Additional file

Additional file 1 **Figure S1** HCV+ group exclusion flowchart. (DOCX 25 kb)

## Data Availability

The dataset from this study is held securely in coded form at ICES. While data sharing agreements prohibit ICES from making the dataset publicly available, access may be granted to those who meet pre-specified criteria for confidential access, available at www.ices.on.ca/DAS. The full dataset creation plan and underlying analytic code are available from the authors upon request, understanding that the computer programs may rely upon coding templates or macros that are unique to ICES and are therefore either inaccessible or may require modification.

## Appendix

**Table 3:** ICD codes used to define chronic conditions

Condition	ICD-O (topographical codes)	ICD-9	ICD-10
**Cancer (any)** (OCR – 10 year lookback)			
Hepatocellular cancer	8170, 8171, 8172, 8173, 8174, 8175		C22.0
**Mental health diagnoses** (10 year lookback for hospitalizations, 2 year lookback for OHIP billings)			
Non-psychotic mood and anxiety disorders^a^		296, 300, 309, 311	F30, F31, F32, F33, F341, F348, F349, F38, F39, F40, F41, F42, F431, F432, F438, F44, F450, F451, F452, F48, F530, F680, F930, F99
Other mental illnesses^a^ (including schizophrenia, delusions, and other psychoses; personality disorders;)		291, 292, 295, 297, 298, 299, 301, 302, 305, 306, 307, 313, 314, 315, 319;	F2, F04, F050, F058, F059, F060, F061, F062, F063, F064, F07, F08, F340, F35, F36, F37, F430, F439, F453, F454, F458, F46, F47, F49, F50, F51, F52, F531, F538, F539, F54, F55, F56, F57, F58, F59, F60, F61, F62, F63, F64, F65, F66, F67, F681, F688, F69, F7, F8, F90, F91, F92, F931, F932, F933, F938, F939, F94, F95, F96, F97, F98
Substance use disorder^a^ (10 year lookback for hospitalizations)		303, 304	F10-F14, F16, F18, F19
Liver failure (one code ever is sufficient for diagnosis)			
Hepatic coma		572.2	
Hepatorenal syndrome		572.4	
Varices with bleeding all diagnoses		456.0	
Esophageal varices – other diseases with bleeding		456.2	
Ascites		789.5	
Peritonitis		567.2	
Hepatic failure NEC			K72
Acute and subacute hepatic failure			K72.0
Acute and subacute hepatic failure without coma			K72.00
Acute and subacute hepatic failure with coma			K72.01
Chronic hepatic failure			K72.1
Chronic hepatic failure without coma			K72.10
Chronic hepatic failure with coma			K72.11
Hepatic failure, unspecified			K72.9
Hepatic failure, unspecified without coma			K72.90
Hepatic failure, unspecified with coma			K72.91
Oesophageal varices with bleeding			I85
Oesophageal varices with bleeding in diseases classified elsewhere			I98.3
Ascites			R18
Peritonitis			K65
Liver transplant (one code ever is sufficient for diagnosis)			
Liver transplant status		V42.7	
Liver transplant status			Z94.4
Complications of liver transplant			T86.4
Liver transplant rejection			T86.41
Liver transplant failure			T86.42
Liver transplant infection			T86.43

^a^uses the ICD codes found in HSPRN multimorbidity SAS macro
