# Correction to: HCV-infected individuals have higher prevalence of comorbidity and multimorbidity: a retrospective cohort study

**DOI:** 10.1186/s12879-019-4579-x

**Published:** 2019-10-24

**Authors:** Curtis L. Cooper, Chrissi Galanakis, Jessy Donelle, Jeffery C. Kwong, Rob Boyd, Lisa Boucher, Claire E. Kendall

**Affiliations:** 1 Department of Medicine, University of Ottawa, Ottawa, Canada; 2 Clinical Epidemiology Program, Ottawa Hospital Research Institute, The Ottawa Hospital-General Campus, G12-501 Smyth Rd, Ottawa, Ontario K1H8L6 Canada; 3 ICES, Toronto, Canada; 4 Department of Family and Community Medicine, University of Toronto, Toronto, Canada; 5 Dalla Lana School of Public Health, University of Toronto, Toronto, Ontario Canada; 6 Sandy Hill Community Health Centre, Ottawa, Canada; 7 Bruyère Research Institute, Ottawa, Canada; 8 School of Epidemiology and Public Health, University of Ottawa, Ottawa, Canada; 9 Department of Family Medicine, University of Ottawa, Ottawa, Canada

**Correction to: BMC Infectious Diseases (2019) 19:712**

**https://doi.org/10.1186/s12879-019-4315-6**

After publication of the original article [1], we were notified that an author’s name has been incorrectly spelled. Jeff Kwong’s full name is Jeffery C. Kwong.
