# Supplementary material for: HCV-infected individuals have higher prevalence of comorbidity and multimorbidity: a retrospective cohort study
Source: BMC Infect Dis. 2019 Aug 23;19:712. doi: 10.1186/s12879-019-4315-6 (PMC6706878; doi:10.1186/s12879-019-4315-6)
Supplement: Supplementary file 1 — Figure S1 HCV+ group exclusion flowchart. (DOCX 25 kb) [file 12879_2019_4315_MOESM1_ESM.docx]

**Supplemental Figure 1: HCV+ Group Exclusion Flow Chart.**

**Total Number of HCV Cases**

(n=2490)

**Final HCV group**

(n=1209)

Invalid visit date or first visit after April 1^st^, 2017 OR

<18 years of age OR

Missing or non-Ontario postal codes OR

Not eligible for OHIP on index date OR

(n=47)

Death date in ICES before index date

(n=351)

Invalid IKN, sex and age values

(n=246)

Not seen by TOHVHP in 5 years prior to April 1^st^, 2017

(n=637)
